# Letter from the Editor in Chief

**DOI:** 10.19102/icrm.2021.120306

**Published:** 2021-03-15

**Authors:** Moussa Mansour


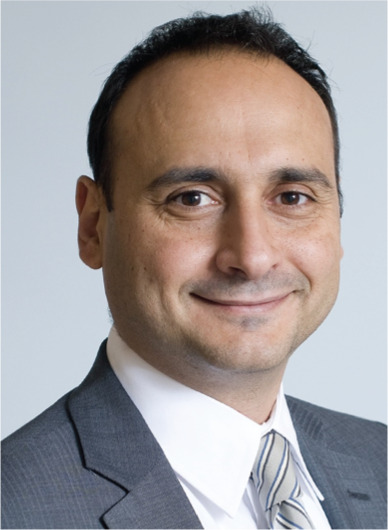


Dear Readers,

From this issue of *The Journal of Innovations in Cardiac Rhythm Management*, I would like to highlight the paper titled “Further Observations on the Use of Pacemakers in Patients with Postural Orthostatic Tachycardia Syndrome with Demonstrated Asystole,” in which Kanjwal et al.^1^ describe outcomes of pacemaker implantation in a subgroup of patients with postural orthostatic tachycardia syndrome (POTS).

The management of patients with POTS can be challenging and the disease can lead to long-term debilitation. Responses to therapy vary and many patients continue to be symptomatic despite receiving multiple treatments. Similar to in patients with neurocardiogenic syncope, a loss of consciousness in POTS is likely the result of a surge in parasympathetic tone and the loss of sympathetic tone, leading to vasodilatation and bradycardia. Also, as is true as well in neurocardiogenic syncope, first-line therapy typically consists of lifestyle changes, followed by the use of medications such β-blockers, fludrocortisone, midodrine, and selective serotonin reuptake inhibitors. In nonresponders, additional medication options such as octreotide, erythropoietin, and pyridostigmine are sometimes considered. Pacemaker implantation in patients with neurocardiogenic syncope has been conducted successfully in many patients. However, some studies, such as the VPS II and Vasovagal Syncope and Pacing (SYNPACE) trials, failed to demonstrate a beneficial role for pacing in this patient population. The benefit of pacemaker implantation in the retrospective study by Kanjwal et al.^1^ is likely due to careful selection of patients and the limiting of pacing to patients with prolonged asystole documented by an implantable loop recorder.

Recently, there has been increased interest in cardioneural ablation for the treatment of cardiogenic syncope. This procedure was first described more than 10 years ago and consists of, first, pace-mapping the autonomic ganglionated plexi, then ablating them. Preliminary results have demonstrated a beneficial effect of this procedure in reducing syncope in patients with severe forms of the disease.^2^ This treatment modality targets the physiologic basis of syncope and has the potential to eliminate the need for pacing in patients who are often young. In this era of advanced mapping and ablation technologies, randomized studies comparing cardioneural ablation to medications and/or pacing are certainly feasible and would provide much-needed data to guide the challenging management of patients with neurocardiogenic syncope and POTS.

Best regards and I hope that you find the content of this issue of *The Journal of Innovations in Cardiac Rhythm Management* beneficial and educational.

Sincerely,


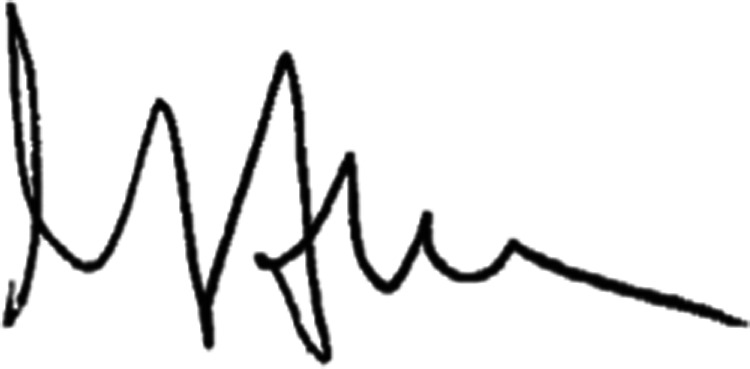


Moussa Mansour, md, fhrs, facc

Editor in Chief

The Journal of Innovations in Cardiac Rhythm Management

MMansour@InnovationsInCRM.com

Director, Atrial Fibrillation Program

Jeremy Ruskin and Dan Starks Endowed Chair in Cardiology

Massachusetts General Hospital

Boston, MA 02114
